# Antioxidant and Antiaging Properties of Agar Obtained from Brown Seaweed *Laminaria digitata* (Hudson) in D-Galactose-Induced Swiss Albino Mice

**DOI:** 10.1155/2022/7736378

**Published:** 2022-02-24

**Authors:** B. S. Reshma, Thabitha Aavula, Vignesh Narasimman, Saravanan Ramachandran, Musthafa Mohamed Essa, M. Walid Qoronfleh

**Affiliations:** ^1^Native Medicine and Marine Pharmacology Laboratory, Department of Medical Biotechnology, Faculty of Allied Health Sciences, Chettinad Academy of Research and Education, Kelambakkam, TN 603 103, India; ^2^Department of Food Science and Nutrition, CAMS, Sultan Qaboos University, Muscat, Oman; ^3^Q3CG Research Institute (QRI), Research & Policy Division, 7227 Rachel Drive, Ypsilanti, MI 48917, USA

## Abstract

The present paper explores the antioxidant and antiaging properties of agar extracted from *Laminaria digitata* (*L. digitata*) on a D-galactose (D-Gal)-induced mouse model. Experimental mice were divided into four groups: group I comprised of control nontreated mice, group II comprised of D-Gal-induced mice, group III mice were treated with extracted agar after D-Gal induction, and group IV mice were given ascorbic acid as a positive control. Antioxidant enzymes and aging marker proteins declined significantly in group II, whereas they were normal in group III and group IV mice. Expressions of interleukin-1*β* (IL-1*β*) in D-Gal-induced mice were significantly enhanced in the liver and brain of the experimental mice, which were otherwise normal in agar-treated mice. Also, IL-6 levels were significantly increased in the liver and reversed in the brain of D-gal mice, while it was regularly in the agar-treated mice. The histopathological analysis of D-Gal-induced mice showed spongiosis and tangles in brain cells, increased fat and decreased collagen contents in the skin, and few dilated sinuses in the hepatic cells. The changes were under control in group III and group IV mice, suggesting the protective effects of agar extracted from *L. digitata* and ascorbic acid.

## 1. Introduction

Aging is a multifactorial natural process of the skin. The changes in the structure and functions of the entire body system are connected to the physical decline associated with aging. Biological aging is rooted in genetics, endocrine, and immune metabolic function factors. Then, environmental influences such as gravity, ultraviolet light exposure, climate, and pollution exacerbate the natural aging process. Several research reports show a correlation between the process of aging and antioxidant functions such as amplified mitochondrial reactive oxygen species (ROS) generation and increased accumulation of oxidant products [1–3]. ROS produced by lipid peroxidation has been implicated in the progressive oxidative deterioration of polyunsaturated fatty acids (PUFAs) of the cell membranes [4]. The ROS originates unstable lipid peroxide species incorporating carbonyl group with the end products, namely, reactive aldehydes such as malondialdehyde (MDA) and 4-hydroxynonenal (HNE), ultimately causing tissue damage [5, 6]. Galactose (Gal) is a monosaccharide that has a similar structure to glucose, differing only in the position of one hydroxyl group. Chronic systemic exposure of rodents to D-Gal causes the acceleration of aging and has been used as an aging agent in experimental models. D-Gal, which is completely metabolized at normal concentrations, is a natural compound in the body. At higher concentrations, however, it converts to aldose and hydrogen peroxide (H_2_O_2_), a reaction catalyzed by galactose oxidase; conversely, superoxide dismutase (SOD) facilitates the transformation of superoxide anions to H_2_O_2_. The overall increase in ROS leads to disruptions in the activities of macromolecules inside the cell [6]. Cosmetic products are used for human skincare by adolescents. The usage intends to maintain the skin in good condition to shield it from the environment's harsh effects and minimize the aging processes [7, 8]. Unfortunately, some of the adverse effects of commercial cosmetics are skin irritation and allergic reactions, swelling, blisters, and surface oozing. On the extreme side, they can cause reproductive toxicity, endocrine disruptions, and cancer to consumers [9, 10]. As a result, consumers are on the lookout for more natural sources of skincare products [11]. Seaweeds are rich sources of polysaccharides used as gelling and thickening agents (e.g., alginates, agar, and carrageenan) including some that have become valuable additives in food manufacturing due to their rheological properties. They are also known to have multiple biological activities, including antioxidant, anticoagulant, antiviral, and immunoinflammatory activities that could find their relevance in nutraceuticals, functional foods, and pharmaceutical applications [12–15]. Previously, the authors have reported on the antioxidant, anticoagulant, and mosquitocidal properties of water-soluble polysaccharides from green, red, and brown seaweeds [16]. However, to the authors' knowledge, there is no report on the antiaging property of agar. *Laminaria digitata* belongs to the phylum Ochrophyta, family Laminariaceae, genus *Laminaria*, and species *digitata*. Hence, the present study was designed and conducted to evaluate the texture, antioxidant, and *in vivo* antiaging properties of agar extracted from the brown seaweed *L. digitata*.

## 2. Methods

### 2.1. Seaweed Collection and Processing

Brown seaweeds (*L. digitata*) were collected from Kanyakumari (Lat 8.0866ºN and Long 77.5544ºE), Tamil Nadu, India. The samples were identified according to the renowned monograph of Marine Biology, Annamalai University, Tamil Nadu, India. First, the seaweeds were washed in seawater, followed by distilled water, and finally were washed with 70% alcohol to remove microflora and other contaminants on the seaweed surface. Finally, the processed seaweeds were shadow dried and stored at −20°C for further study.

### 2.2. Extraction of Aqueous Polysaccharide

The dried seaweed samples were blended and ground into a fine powder using a blender. A 5 g of powdered seaweeds was dissolved in 100 ml of Milli-Q water and then autoclaved at 121°C for 1 hour. The water-soluble polysaccharides were filtered through a Whatman no. 1 filter paper to remove debris in the extract and further centrifuged at 5000xg for 15 min at room temperature to eliminate any particulate matter. The solution was kept at room temperature until gelation. The gelated agar was frozen at room temperature overnight. After bleaching, the samples were dried at 60°C overnight [17].

### 2.3. Bleaching of Agar

The gelated agar seaweed samples were washed with sodium hypochlorite (NaClO). The washing was repeated up to 5 times. After NaClO treatment, it was diluted with H_2_O_2_. The color change has been noted as dark brown to pale yellow color [18].

### 2.4. Food Texture Analyses

The texture parameters of the extracted agar from *L. digitata* were measured using the TA.XTplus Texture Analyzer (Stable Microsystems, UK) in back extrusion mode and constituted with a 36 mm cylinder probe. The parameters were set as follows: data acquisition rate: 200 PPS, test speed: 1.0 mm/s, load cell: 5 mm, and distance: 40 mm. The agar from *L. digitata* was loaded onto the analyzer platform, and the probe was allowed to be flush with the surface. The extract holders were supported by a heavy-duty rig plate attached to the crosshead of the instrument. Upon data acquisition, it was plotted as a graph. The peaks on the graph represent the force at which the probe reaches a maximum penetration depth of 75% of the original agar height. A relaxation of the sample over the two-second holding period resulted in subsequent decay in force, followed by the withdrawal of the probe to a tracking force of 5 g, thus allowing the sample to “spring back”. The maximum force (*g*) required to penetrate the product was recorded as the hardness, adhesiveness, cohesiveness, springiness, and gumminess of agar from *L. digitata*. The experiments were run in triplicate, and an average value was statistically reported [19].

### 2.5. In Vitro Antioxidant Activity

#### 2.5.1. 2,2-Diphenyl-1-picrylhydrazyl (DPPH) Scavenging Activity

The DPPH radical scavenging assay was performed using a 96-well microtiter plate. A 50 µl of 100 µg/ml methanolic DPPH solution was added to 200 µl of different concentrations of agar sample (12.5–100 µg/ml) and mixed with a vortex mixer. Ascorbic acid was used as a standard [20].

#### 2.5.2. H_2_O_2_ Scavenging Activity

The scavenging activity of the agar was determined. A 0.3 ml agar solution was reacted with 600 µl H_2_O_2_ 40 mM solution. Then, it was allowed to stand for 10 min at room temperature. The absorbance of the reaction mixture was measured at 230 nm by a UV spectrophotometer [21].

#### 2.5.3. Total Antioxidant Capacity

The total antioxidant activities of the agar from the seaweeds were determined. A 0.3 ml agar sample was reacted with 3 ml total antioxidant capacity reagent solution (0.6 M H_2_SO_4_, 28 mM sodium phosphate, and 4 mM ammonium molybdate). The reaction mixture was incubated in a water bath at 95°C for 90 min. The absorbance of the reaction mixture was measured at 695 nm by a UV-visible spectrophotometer [21].

#### 2.5.4. Phosphomolybdate Activity

The antioxidant activity of agar was evaluated by the green phosphomolybdenum complex formation protocol. A reaction mixture solution was prepared by adding 0.588 ml of sulfuric acid (H_2_SO_4_), 0.049 g ammonium molybdate, and 0.036 g sodium phosphate. The volume of the final solution was made up to 10 ml with distilled H_2_O. Afterwards, 10 mg of agar was dissolved in 1 ml of dimethyl sulfoxide (DMSO). A 100 *μ*l from the prepared agar sample was taken, and 1 ml of the reagent mixture solution was added to it and then incubated in a boiling water bath at 95°C for 90 min. After 90 min, the absorbance of the solution was read at 695 nm. Ascorbic acid (10 mg/ml DMSO) was used as a standard [22].

#### 2.5.5. Ferric Reducing Antioxidant Power Assay (FRAP Assay)

The working FRAP reagent was produced by mixing 300 mM acetate buffer (pH 3.6), 10 mM 2,4,6-tripyridyl-s-triazine (TPTZ) solution, and 20 mM FeCl_3_.6H_2_O in a 10 : 1:1 ratio before use and was heated to 37°C in a water bath for complete dissolution. The blank reading was measured by adding 200 *μ*l of this working solution to the microtiter plate and read at 593 nm using a UV spectrophotometer. The sample reading was calculated by adding 3 ml of the freshly prepared working solution with 100 *μ*l of agar extracts and 300 *μ*l of distilled water and then left for 4 min at room temperature before reading absorbance at 593 nm using a UV spectrophotometer. Here, ferrous sulfate (FeSO_4_) was used as standard and for calibration in the concentrations of 0 to 1000 *μ*M [23].

### 2.6. Behavioral Studies

Antiaging was experimentally induced in female Swiss albino mice following the ethical clearance obtained from Institutional Animal Ethics Committee (IAEC4/Alr.No. 23/dated 12.12.17). Extracted agar (100 mg/kg) was suspended in 0.1 M phosphate-buffered saline (PBS) and administered orally using an 18 mm gauge for 42 days. Control mice received regular food and water *ad libitum*. Ascorbic acid (100 mg/kg)-treated mice served as the positive control. The animals were divided into four groups (Table 1). Food and water intake of all mice groups were measured up to 42 days. The behavioral parameters such as locomotor function, light-dark, hot plate, and rotarod test of experimental mice groups were determined on 0, 2^nd^, 4^th^, and 6^th^ weeks [24]. At the end of the experimental period, blood was collected using a 1 ml syringe from the tail vein of the mice. The mice were then sacrificed by halothane inhalation. The liver, brain, kidney, and portions of the skin were dissected, fixed in formalin, and embedded in paraffin, and the morphological changes were detected under the microscope. The rest of the dissected organs were stored in PBS and stored at −20°C for further use.

#### 2.6.1. Locomotor Activity

Locomotor activity was evaluated by placing a mouse into the center of a clear Plexiglas (40 × 40 × 30 cm) open-field arena and allowing the mouse to explore for 5 min. Bright overhead lighting was approximately 500 lux inside the arenas, while noise was present at about 60 dB (INCI Photoactometer, India). The total distance (locomotor activity), movement time (sec), movement speed (cm/sec), and center distance (the distance traveled in the center of the arena) were recorded manually. The center distance was divided by the total distance to obtain a center distance-to-total distance ratio. The center distance-to-total distance ratio can be used as an index of anxiety-related responses. Data were collected at 2, 3, 4 and 5 min intervals over the session [25].

#### 2.6.2. Light-Dark Exploration Test

Experimental mice were put through the light-dark exploration test, which consists of a polypropylene chamber (44 × 21 × 21 cm) unequally divided into two chambers by a black partition containing a small opening. The large chamber was open and brightly illuminated (800 lx), while the small chamber was closed and dark. White noise was present in the room at approximately 55 dB in the test chamber. Mice were placed on the illuminated side and allowed to move freely between the two chambers for 5 min [26].

#### 2.6.3. Hotplate Test

The hotplate test was used to evaluate sensitivity to a painful stimulus. Mice were placed on a 55.0°C (±0.3) hotplate, and the latency to the first hind-paw response was recorded. The hind-paw response was either a foot shake or a paw lick [27].

#### 2.6.4. Rotarod Test

Mice were placed on a rotating rod (INCI Rotarod, India) that accelerated from 4 to 40 rpm. For two consecutive days, four trials were performed per day with 45–60 min intervals between trials. The maximum duration of each trial was 5 min. The time at which the mice fell off the rod was recorded [28].

### 2.7. In Vivo Antiaging Marker Studies

#### 2.7.1. Collagen Measurement

The amount of collagen in the dermis was measured using a Sircol Collagen Assay Kit based on the fact that the Sirius red dye binds to the side chains of the amino acids in collagen [29].

#### 2.7.2. Elastin Measurement

The amount of elastin in the dermis was measured using a Fastin Elastin Assay Kit based on the fact that the Fastin dye binds to the side chains of the amino acids in elastin [29].

#### 2.7.3. Protein Extraction

The frozen skin samples were minced and homogenized in 10 volumes of extraction buffer Tris-HCl (pH 7.8). The homogenate was centrifuged at 9,000xg at 4°C for 15 min, and the supernatant was collected as an extracted protein. The protein concentration of the supernatant was measured by Lowry's method using a DC Protein Assay Kit [29].

#### 2.7.4. Skin Aging Assay

The levels of elastin, collagen, hyaluronidase, and tyrosinase in skin tissues were examined using the kits procured from Cayman Chemicals, USA [30].

### 2.8. Biochemical Analysis and Histological Analysis

The blood and tissue levels of reduced glutathione (GSH), hydroxyproline, malondialdehyde (MDA), total protein, and the activities of total SOD, catalase (CAT), and total antioxidant capacity (T-AOC) in the blood and tissues were determined using commercial kits (Sigma, USA). The brain, liver, kidney, and skin of each group of mice were fixed in 10% formalin for 24 h; tissues and dorsal skin samples were dehydrated in various ways before being implanted in paraffin, sectioned thinly, dewaxed, and stained with hematoxylin-eosin [31]. Cross sections were selected from three plates per sample, and the morphological changes were examined under the microscope.

### 2.9. RNA Extraction and Quantitative PCR

A 100 mg of the brain tissue sample was taken, and 1 ml of TRIzol (Sigma, USA) was added and homogenized. The solution was incubated at room temperature for 30 min and then centrifuged at 12,000 rpm for 10 min. The supernatant was transferred, and after 5 min, about 200 *μ*l of chloroform was added, mixed, and incubated at room temperature for 15 min. Then, it was followed by centrifugation at 12,000 rpm for 10 min at 4°C. The upper aqueous layer was transferred, and a 500 *μ*l of isopropanol was added and then incubated at room temperature for 10 min and finally centrifuged at 12,000 rpm for 10 min at 4°C. To the pellet, a 500 *μ*l of 70% ethanol was added, washed (5000 rpm for 5 min at 4°C), and then air-dried. The pellet was then dissolved in 20 *μ*l of diethyl pyrocarbonate (DEPC)-treated water. The RNA was quantified using a UV spectrophotometer by measuring the ratio of absorbance at wavelengths 260 nm and 280 nm (NanoDrop, Thermo Scientific, USA).

### 2.10. Statistical Analysis

Each test was performed on six mice, and all results were expressed as the mean ± SD. A *P* value of <0.05 was considered significant. Statistical analysis was performed using the IBM SPSS Statistics for Windows, version 22 (IBM Corp., Armonk, N.Y., USA).

## 3. Results and Discussion

Aging is an intricate progression that disturbs the tasks of all organs and tissues and most often has an irreversible impact on their mechanical behavior. The most significant noticeable effects of aging are perceived in the skin and have been broadly investigated for medical and cosmetic resolutions. From a biological appearance, aging is a predictable natural process [24].

About 5 kg of the selected brown seaweed, *L. digitata* was collected from the Kanyakumari coast, Tamil Nadu, India, and processed. After being shadow dried, the dry weight and the yield of agar from *L. digitata* were found to be 266.66 g and 20%, respectively. This yield of agar from *L. digitata* was high when compared to prior results [32]. It has been documented that the difference in agar yield varies from species to species; also, it is dependent on the harvested season. The study results were in accordance with the report of Armisen et al. [33] who pointed out that the yield of agar varied between species and geographical areas of collections.

The proximate biochemical composition and food texture analysis of *L. digitata* are shown in Table 2. The moisture content of the *L. digitata* was reported to be 22.18%, and among the selected macroelements, sodium (Na) was found to be maximum (1.74%) while phosphorous (P) was found to be minimum (0.03%). Further, the carbon and phosphorous ratio (C : P) was found to be 0.72% of the sample of harvested brown seaweed *L. digitata*. Moreover, among the selected microelements, the content of copper was observed at maximum (73.41 ppm), and cobalt was recorded at minimum (2 ppm). The elemental content of agar from *L. digitata* was higher when compared to the report of Fleuerence et al. [34]. The difference in the mineral content could be attributed to the species difference and the method selection for quantification. The overall biochemical analyses findings were in agreement with Fleuerence et al. [34] who had mentioned that the biochemical concentration of seaweed varies according to the factors stated above. The texture properties such as hardness (234.43 ± 0.60 g), adhesiveness (50.61 ± 0.40 N/mm), cohesiveness (0.825 ± 0.11), springiness (3.250 ± 0.12), and gumminess (2.969 ± 0.10) of agar from *L. digitata* were maximum when compared to the texture profile analysis of the starch gel prepared from corn [35]. The difference in the texture analysis was due to the monosaccharide composition differences between the species [36], and the results support the present finding that aqueous extract can be easily degraded due to the presence of monomeric chains [37].

Antioxidant activity was determined from the brown seaweed *L. digitata*. The methods differ in terms of their assay and experimental conditions. The *in vitro* antioxidant activity of agar from the brown seaweed *L. digitata* showed higher activity at higher concentrations [35]. The DPPH scavenging activity in agar from brown seaweed was assayed and is presented in Table 3. The 200 µl concentration of agar showed significantly elevated levels of scavenging activity (83.70 ± 3.83%) (*P* < 0.05) when compared to the concentration of 50 µl (32.68 ± 1.21%). The standard 100 µl of ascorbic acid was at (62.42 ± 1.11%) DPPH scavenging activity. Hence, the significant variations in the DPPH scavenging abilities of the bioactive compounds isolated from seaweed might be due to variations in the type of extraction and/or differences in the zone of collection of the seaweeds [35].

The H_2_O_2_ radical scavenging activity of agar extracted from brown seaweed is shown in Table 3. The agar exhibited increased H_2_O_2_ scavenging activity (89.85 ± 3.86%) at a concentration of 200 µl, closely followed by the concentrations of 100 µl (64.99 ± 1.93%) and 50 µl (47.47 ± 0.97%), and the difference was observed to be significant (*P* < 0.05). Ascorbic acid at 100 µl displayed H_2_O_2_ radical scavenging activity of 80.82 ± 3.53%, resulting in complete buffering of the free radicals that might be generated. The results of the present investigation seemed higher when compared to the H_2_O_2_ radical scavenging activity of the aqueous extract of Indian seaweed [38]. The observed differences in the percentage of H_2_O_2_ scavenging activity might be attributed to the variations in the geographical areas of collection from the sea or season of harvesting. It might also be due to the differences in extraction procedures, like using different solvents and filtration procedures.

The total antioxidant activity of agar extracted from brown seaweed is shown in Table 3. The agar significantly showed increased total antioxidant activity (67.03 ± 3.30%) (*P* < 0.05) at a concentration of 200 µl, followed by concentrations of 100 µl (33.49 ± 2.14%) and 50 µl (16.77 ± 1.08%), respectively. The phosphomolybdate activity of agar extracted from brown seaweed is given in Table 3, which is caused by the reduction of Mo^6+^ ions to form a green phosphate/molybdenum V complex. The agar from *L. digitata* was significantly increased (*P* < 0.05) by the scavenging phosphomolybdate assay (66.48 ± 3.03%) at a concentration of 200 µl and followed by concentrations of 100 µl (33.21 ± 2.05%) and 50 µl (16.63 ± 2.92%), correspondingly. The standard ascorbic acid exhibited (75.18 ± 3.54%) scavenging phosphomolybdate activity, resulting in complete buffering of the free radicals that might be generated.

The potency reduction activity of agar extracted from the selected brown algae was calculated, and the result was expressed as a percentage. The potency reduction activity of FRAP is estimated by measuring the Fe_3_^+^-Fe_2_^+^ transformation in the presence of agar extracted from *L. digitata*. The selected brown seaweed exhibited increased FRAP reducing power activity (72.03 ± 3.21%) at 200 µl followed by (82.31 ± 3.78%) at 100 µl (*P* < 0.05). The antioxidant activity is brought about by the ability to scavenge free radicals, and thus, in the current study, the agar of brown seaweed showed good antioxidant activity by scavenging ROS.

**Figure 1:**
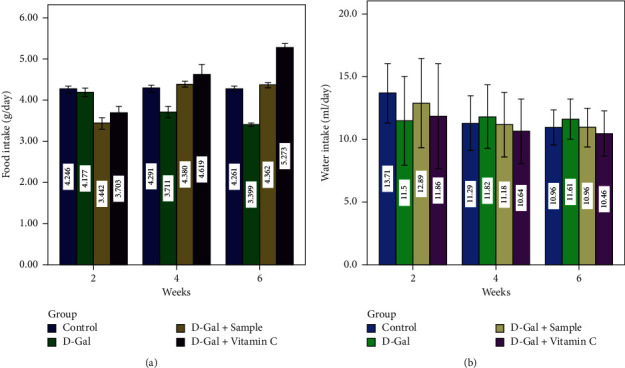
Food and water intake of experimental mice.

**Figure 2:**
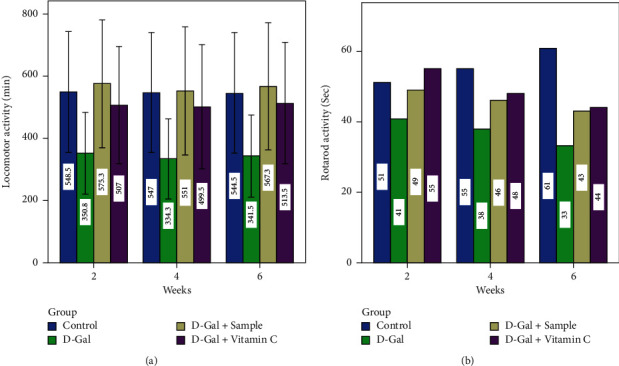
Locomotor and rotarod activities of experimental mice.

Animal experiments revealed significant variations in food and water intake (Figures 1(a) and 2(b)) and body weight change (Table 4), in the D-Gal (group II)-injected mice when compared with the control group (group I) (*P* < 0.05). The water intake was significantly lower in D-Gal-injected mice when compared to group I mice in the second week (*P* < 0.05). However, it significantly increased in the subsequent weeks (*P* < 0.05). Prior to the animals being sacrificed, no mice died during the entire experiment. However, at the beginning of the second week, mice demonstrated noticeable effects of D-Gal aging, such as apathy, delayed response, and slow motion, which continued for up to six weeks. Furthermore, group II mice had a significantly higher body weight than group I mice (*P* < 0.05).

**Figure 3:**
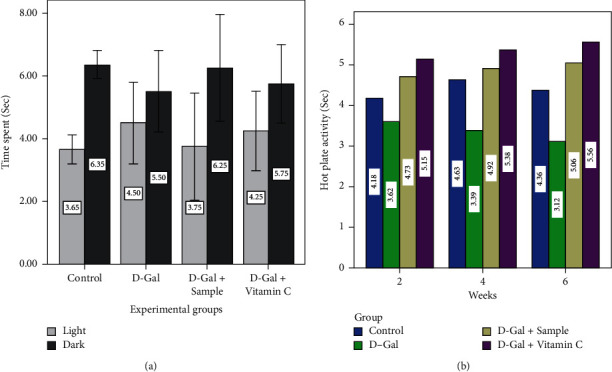
Light and dark and hotplate activities of experimental mice.

Current outcomes were endorsed by Swallow et al. [37] who claimed that food consumption and lack of physical activity played a major role in body composition. For the locomotor and rotarod (Figure 2) and light and dark and hot plate activities (Figure 3), group II mice experienced a significant decline when compared to group I mice in early (0 weeks) and late (6 weeks) (*P* < 0.05). The results of the present study suggested that all the abovementioned activities clearly proved that aging was induced in the D-Gal (group II) mice. On the other hand, the characteristics in the control sample (group III) and in the ascorbic acid orally administered mice (group IV) were found to be normal.

Figures 3(a) and 3(b) present the light and dark exposure and hot plate activity of experimental mice. According to the data, the time spent in light and dark in group II mice was significant (*P* < 0.05) compared to group I mice during the 6th week. The response of hot plate activity of group II mice was lower than that of group I mice during the second week onwards, and it was further decreased at the end of the experiment period (6^th^ week) (*P* < 0.05). The results strongly suggested that all the abovementioned activities clearly proved that aging was induced in the D-Gal (group II) mice, while these behaviors were stabilized in the control sample (group III) and in the ascorbic acid coadministered mice (group IV) [38]. It was also suggested that behavioral activities were slow in the aging mice. It slows down the rate of aging by influencing the basic cellular metabolism of motor activity at the organism level [24].

**Figure 4:**
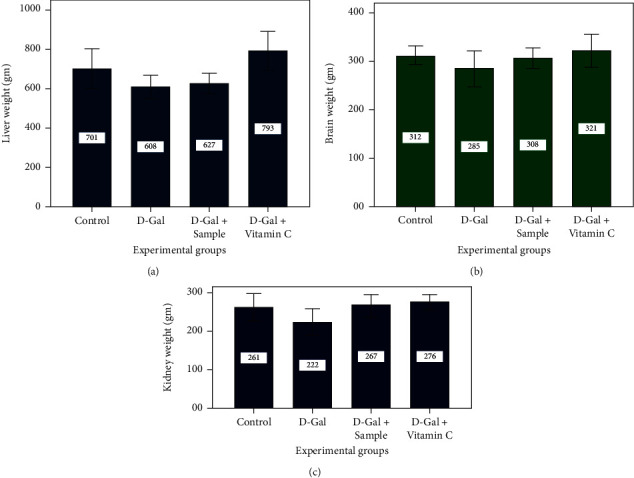
Brain, liver, and kidney weight of experimental mice.

Mice injected with D-Gal showed noticeable symptoms of aging. The organ indices in the brain, liver, and kidney of the model group were lower than those of the control group as depicted in Figures 4(a) and 4(c) (*P* < 0.05). With age comes functional weakening and tissue/organ degeneration. Mostly, changes in the organs such as the brain, liver, and kidney weight were evident. So, the change in organ index is a significant sign of organism senescence. D-Gal could induce physical aging and diminish organ indices of the brain, liver, and kidney in group II, according to the present experimental data. In group II, agar from brown seaweed could efficiently recover all organ indices. It demonstrated that seaweed agar could play a role in preserving the weight of all these organs from declining in the D-Gal experimental mouse model. With increased lifespan comes functional decline and atrophy of all organs and tissues. Differences in brain and kidney mass were noticeable [39]. Consequently, an organ index change was an essential sign of creature senescence, proving that the extracted agar and ascorbic acid could play a vital role in protecting the weight of those organs from decreasing [40].

**Figure 5:**
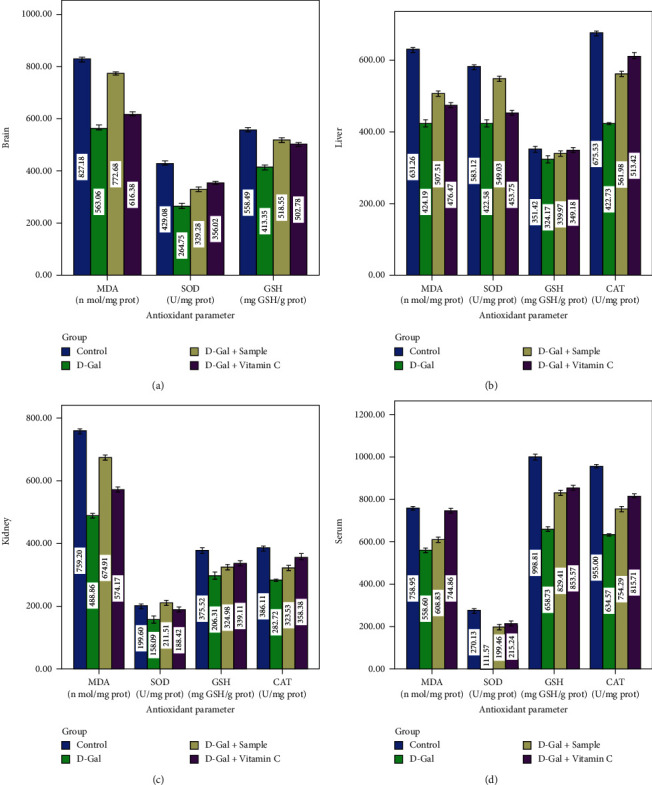
*In vivo* antioxidant activity of experimental mice.

**Figure 6:**
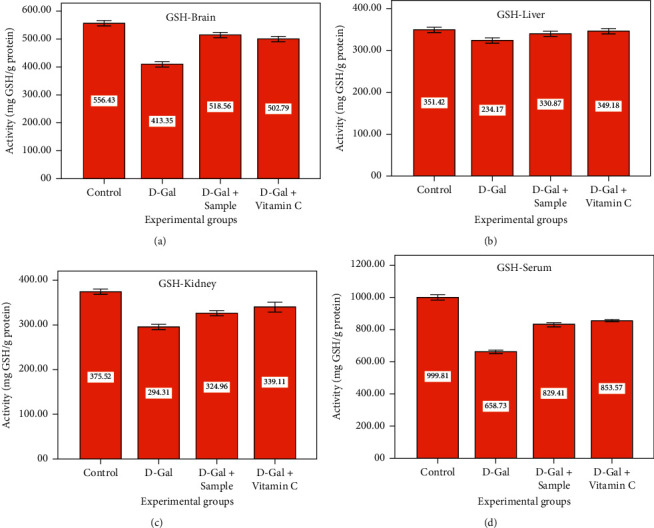
GSH level of experimental groups.

The *in vivo* antioxidant defense systems such as MDA, SOD, CAT, and GSH in mice administered with D-Gal (group II) exhibited a significant decrease in the tissues of the liver, brain, and kidney when compared to the control group I mice (*P* < 0.05). Group III mice showed a significant increase in the activities of all of these parameters (*P* < 0.05, Figures 5(a)–5(d)). Cell life in an oxygenated background has necessitated the progression of effective cellular tactics to identify and detoxify metabolites of ROS. The effects mentioned above could significantly disturb a host of physiological practices and metabolic pathways contributing to the aging of the animal and skin. For example, excess ROS can cause lipid peroxidation *in vivo*. One of the final oxidation products is MDA, which induces cytotoxicity. Therefore, the content of MDA might indicate the body's level of lipid peroxidation [19] and reveal the extent of cell injury carried out by ROS. The rise in the activities of the primary enzymatic antioxidant defenses—MDA, SOD, CAT, and GSH—in the sample coadministered group (group III) and vitamin C (group IV) mice was significant (*P* < 0.05). Presumably small amounts of agar may enhance the antioxidant activity of SOD. The GSH activity of the brain, liver, kidney, and serum (Figures 6(a)–6(d)) of D-Gal-induced (group II) mice was significantly lower than that of the control, the sample, and vitamin C administered groups of mice (*P* < 0.05). The present findings were supported by the report of Ye et al. [24] who suggested that ethyl acetate extract from the plant (*I. polycarpa* defatted fruit) increased the antioxidant levels in experimental mice. The GSH level is a significant factor for quantifying the level of antioxidative activity *in vivo* and has tremendous antioxidant and detoxifying activities. It can protect almost every cell in the body. GSH not only eradicates free radicals *in vivo* but also boosts the organism's immunity level [41].

**Figure 7:**
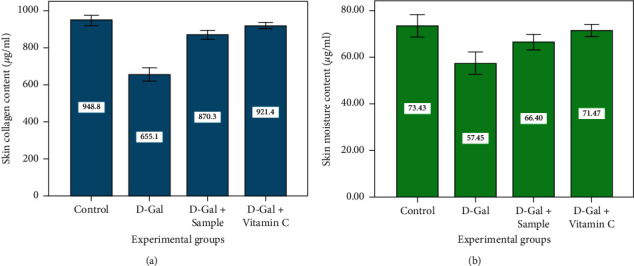
Elastin and moisture contents of experimental mice.

All the parameters in behavioral studies and the organ indices of D-Gal-induced aging mice were reduced. The antioxidant enzymes, elastin, collagen, and hyaluronic acid levels were low in D-Gal-induced mice, which endorsed the role of D-Gal on metabolic age changes in the skin. At the same time, all these levels were high in agar-treated mice. Further, the skin's moisture content affects the skin's physiological function. These expressions (IL-1*β* and IL-6) were altered in D-Gal-induced mice which were brought to a normal level by mice administered with agar. The loss of elastin and moisture caused by the D-Gal-induced mice group II was significantly lower than that of control mice (group I) (*P* < 0.05), indicating that elastin and moisture would progressively alter with the age of the skin. Agar-administered group III mice had significantly increased elastin content when compared to group II mice (*P* < 0.05, Figures 7(a) and 7(b)).

**Figure 8:**
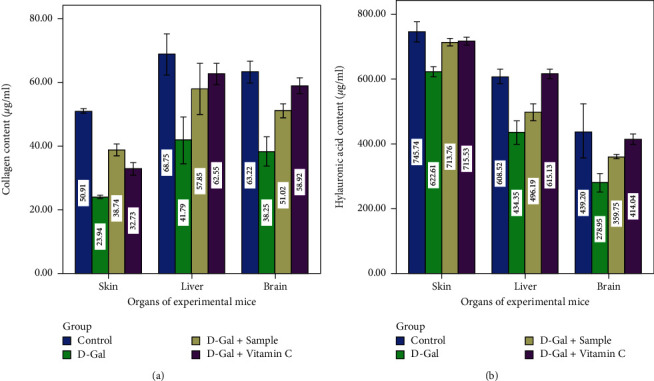
Collagen and hyaluronic acid contents of experimental mice.

The present results were concurrent with the report of Ye et al. [24] who indicated that D-Gal reduced these cytokine factors, which were normalized by the ethyl acetate extract of *I. polycarpa* defatted fruit. Many scientific experts pointed out that cosmetic usage makes women lose elastin quicker than men [42]. Collagen fiber and hyaluronic acid are critical components of the skin. Collagen and hyaluronic acid content in group II mice decreased significantly relative to group I mice (*P* < 0.05). The variation in the amount of collagen can therefore accelerate skin aging (Figures 8(a) and 8(b)). Fittingly, brown algae agar improved the condition of mouse skin due to its high moisture and element content. Brown algae agar can effectively enhance the antioxidant activity, maintain collagen, elastin, and hydration, and reduce the MDA content of aging mouse skin. The morphological modifications of mouse skin have demonstrated the antiaging activity of agar. Collagen fibers decrease significantly with age and therefore make the skin inflexible and saggy. Thus, modifying the collagen content can accelerate the aging process of the skin [42].

**Figure 9:**
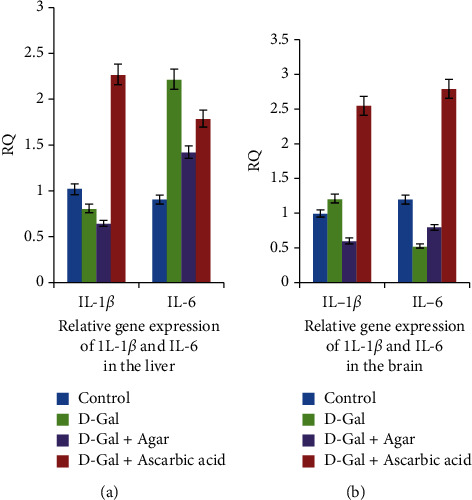
Relative quantification (RQ) of gene expression of IL-1*β* and IL-6 in the liver and brain of experimental groups of mice.

**Figure 10:**
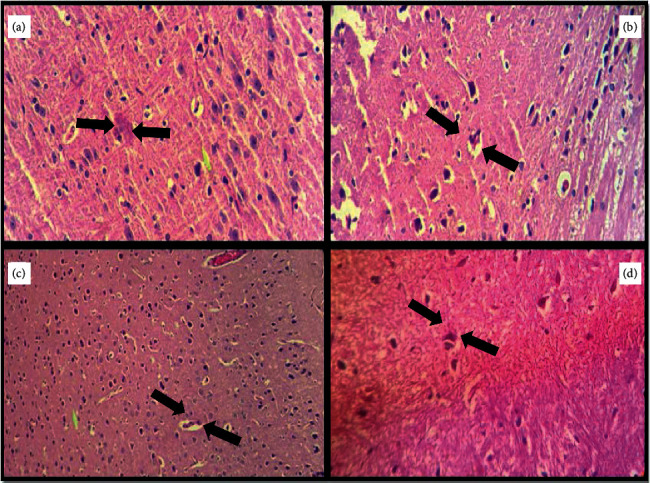
Histological evaluation of the brain of experimental group mice.

**Figure 11:**
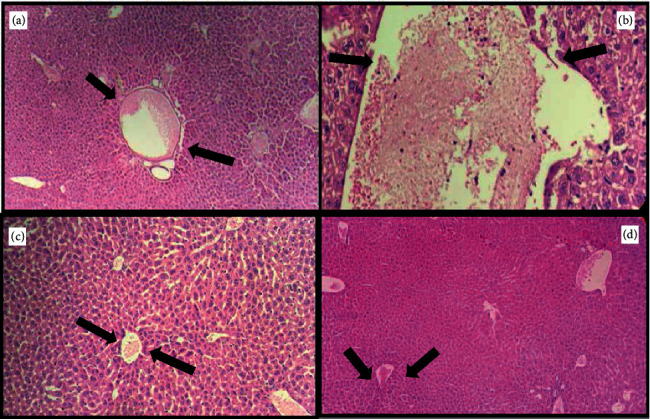
Histological evaluation of the liver of experimental group mice.

**Figure 12:**
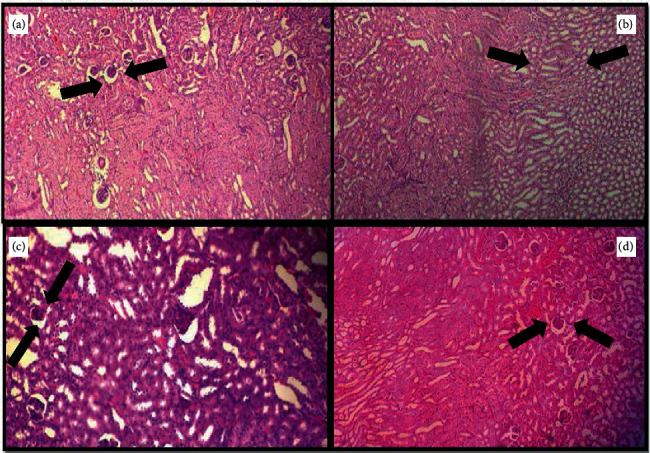
Histological evaluation of the kidney of experimental group mice.

**Figure 13:**
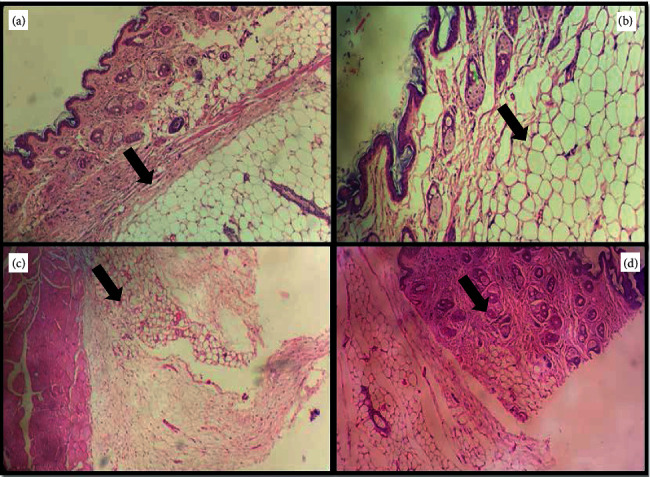
Histological evaluation of the skin of experimental group mice.

The effect on IL-1*β* and IL-6 in the experimental groups is shown in Figure 9. The D-Gal-treated group exhibited increased IL-1*β* and IL-6 gene expression in the brain (*P* < 0.001 and *P* < 0.01, respectively); meanwhile, they only increased IL-1*β* gene expression in the liver (*P* < 0.01). In mice treated with D-Gal + agar 100 mg/kg, the IL-1*β* and IL-6 gene expression in the brain were inhibited, when compared with the D-gal-treated group alone. Further, IL-1*β* gene expression in the liver decreased conspicuously in the D-Gal + agar-treated group of mice. The present study reinforced the finding that D-Gal induced noticeable aging and oxidative stress in the brain and liver of experimental animals [24]. The paraffin sections of the brain, the liver, the kidney, and the skin of experimental mice are presented in Figures 10–13. It was noticed that the brain cell number of group II was less than that of groups I, III, and IV. Typically, cells in the brain degenerate with age, much like the reductions seen in group II mice. However, this number was regenerated in the agar-treated (group III) and vitamin C (group IV)-treated mice. In D-Gal-injected mice, hepatocytes were considerably damaged, displaying few appearances of ballooning degeneration and becoming shallow in comparison with group I mice. Further, all these changes were normalized in the sample-administered and ascorbic acid-treated mice. Similar histopathological findings were also reported by Ye et al. [24] who indicated that morphological alterations were seen in organs such as the brain, liver, and skin of the aging mice that administered D-Gal.

## 4. Conclusions

The in vitro antioxidant activity of agar from the brown algae *L. digitata* showed higher potency at higher concentrations. All parameters in the behavioral studies and organ indices for aging were reduced in the D-Gal-induced mice. Antioxidant enzymes, elastin, collagen, and hyaluronic acid levels were weak in D-Gal-induced mice, suggesting that D-Gal modified the metabolic age of the skin. All the parameters were elevated in mice exposed to agar. The expression of IL-1*β* and IL-6 was altered in D-Gal-induced mice, which was in turn brought back to normal by mice administered with agar. Finally, the anti-skin aging properties of agar were also confirmed by histopathology. All the outcomes suggested that agar from *L. digitata* holds promise as an antiaging natural phytomedicine and skincare product in the future.

## Figures and Tables

**Table 1:** Experimental groups of mice.

Group 1	Normal mice (normal diet and water)
Group 2	Aging-induced mice (100 mg/kg D-Gal; s.c. injection)
Group 3	Aging-induced (100 mg/kg; s.c.) mice + 100 mg/kg of agar (orally)
Group 4	Positive control mice (100 mg/kg D-Gal; s.c.) + ascorbic acid 100 mg/kg (orally)

**Table 2:** Proximate composition and food texture analysis of agar from *L. digitata.*

Proximate analysis															
Brown seaweed	Moisture (%)	C : P (%)	Ca (%)	P (%)	Na (%)	K (%)	Mn (%)	Zn (%)	Fe (%)	Cu (%)	Co (%)
*L. digitata*	22.18	0.72	0.1	0.03	1.74	0.99	14.50	40.50	29.00	73.41	2.00

Food texture															
Hardness (g)	Adhesiveness (N/mm)	Cohesiveness				Springiness				Gumminess			

243.43 ± 0.6	50.61 ± 0.40	0.825 ± 0.11				3.250 ± 0.12				2.969 ± 0.10			

**Table 3:** *In vitro* antioxidant activity in agar from brown seaweed.

S. no.	Parameters	Agar (50 µl)	Agar (100 µl)	Agar (200 µl)	Ascorbic acid (100 µl)
1.	DPPH scavenging activity (%)	32.68 ± 1.21	65.33 ± 2.41	^*∗*^83.70 ± 3.83	^*∗*^62.42 ± 1.11
2.	H_2_O_2_ scavenging assay (%)	47.47 ± 0.97	64.99 ± 1.93	^*∗*^89.85 ± 3.86	^*∗*^80.82 ± 3.53
3.	T-AOC (%)	16.77 ± 1.08	33.49 ± 2.14	^*∗*^67.03 ± 3.30	^*∗*^72.28 ± 3.73
4.	Phosphomolybdate assay (%)	16.63 ± 2.92	33.21 ± 2.05	^*∗*^66.48 ± 3.03	^*∗*^75.18 ± 3.54
5.	FRAP assay (%)	20.51 ± 1.30	41.05 ± 2.61	^*∗*^72.03 ± 3.21	^*∗*^82.31 ± 3.78

**Table 4:** Body weight of experimental mice.

S. no.	Weeks	Group I (g)	Group II (g)	Group III (g)	Group IV (g)
1.	0	^*∗*^26.17 ± 2.36	^*∗*^29.50 ± 2.29	27.33 ± 1.42	28.67 ± 1.05
2.	2	^*∗*^28.67 ± 2.91	^*∗*^29.50 ± 2.50	26.33 ± 1.49	28.00 ± 1.29
3.	4	^*∗*^34.33 ± 1.50	^*∗*^36.83 ± 2.01	31.00 ± 1.53	31.67 ± 1.67
4.	6	^*∗*^27.33 ± 1.31	^*∗*^39.83 ± 1.35	31.00 ± 1.53	31.33 ± 1.33

Statistical significance: *P* < 0.05 (DMRT). ^*∗*^Comparisons were made between groups I and II.

## Data Availability

All data generated or analyzed during this study are included in this published article.

## Supplementary Materials

Supplementary Materials In the early (0 weeks) and late (6 weeks), group II mice showed a significant decrease in light and dark activity (Figure S1) as compared to group I mice (*P* < 0.05). All of the abovementioned activities clearly indicated that ageing was induced in the D-Gal (group II) mice, according to the findings of this study. When compared to D-Gal, there are no significant changes (shrinkage) in the skin of the experimental mice (group I), as shown in Figure S1 (group II). However, in the agar-treated (group III) mice, this change was reversed (Figures S2–S4). Statistical significance: *P* < 0.05 (DMRT). ^*∗*^Comparison was made between 200 µl of sample and standard.
