# Supplementary material for: Antioxidant and Antiaging Properties of Agar Obtained from Brown Seaweed Laminaria digitata (Hudson) in D-Galactose-Induced Swiss Albino Mice
Source: Evid Based Complement Alternat Med. 2022 Feb 24;2022:7736378. doi: 10.1155/2022/7736378 (PMC8894001; doi:10.1155/2022/7736378)
Supplement: Supplementary Materials — In the early (0 weeks) and late (6 weeks), group II mice showed a significant decrease in light and dark activity (Figure S1) as compared to group I mice (P < 0.05). All of the abovementioned activities clearly indicated that ageing was induced in the D-Gal (group II) mice, according to the findings of this study. When compared to D-Gal, there are no significant changes (shrinkage) in the skin of the experimental mice (group I), as shown in Figure S1 (group II). However, in the agar-treated (group III) mice, this change was reversed (Figures S2–S4). Statistical significance: P < 0.05 (DMRT). ∗Comparison was made between 200 µl of sample and standard. [file 7736378.f1.docx]

**Antioxidant and anti-aging properties of agar obtained from brown seaweed**

***Laminaria digitata* (Hudson) in D-galactose induced Swiss albino mice**

B. S. Reshma^1^, Thabitha Aavula^1^, Vignesh Narasimman^1^, Saravanan Ramachandran^1*^, Musthafa Mohamed Essa^2^ and M. Walid Qoronfleh^3^

^1^Native Medicine and Marine Pharmacology Laboratory, Department of Medical Biotechnology, Faculty of Allied Health Sciences, Chettinad Academy of Research and Education,
Kelambakkam - 603 103, Tamil Nadu, India

^2^Department of Food Science and Nutrition, CAMS, Sultan Qaboos University, Muscat, Oman

^3^Q3CG Research Institute (QRI), Research & Policy Division, 7227 Rachel Drive, Ypsilanti,
MI 48917, USA

**^*^ Corresponding author:**

**Dr. R. Saravanan**

**Native Medicine and Marine Pharmacology Laboratory**

**Faculty of Allied Health Sciences**

**Chettinad Academy of Research and Education,**

**Kelambakkam - 603 103, Chengalpattu (Dist)**

**Tamil Nadu, India.**

**E-mail:** [**saran_prp@yahoo.com**](mailto:saran_prp@yahoo.com)

**Phone: +91 44 4741 9038**


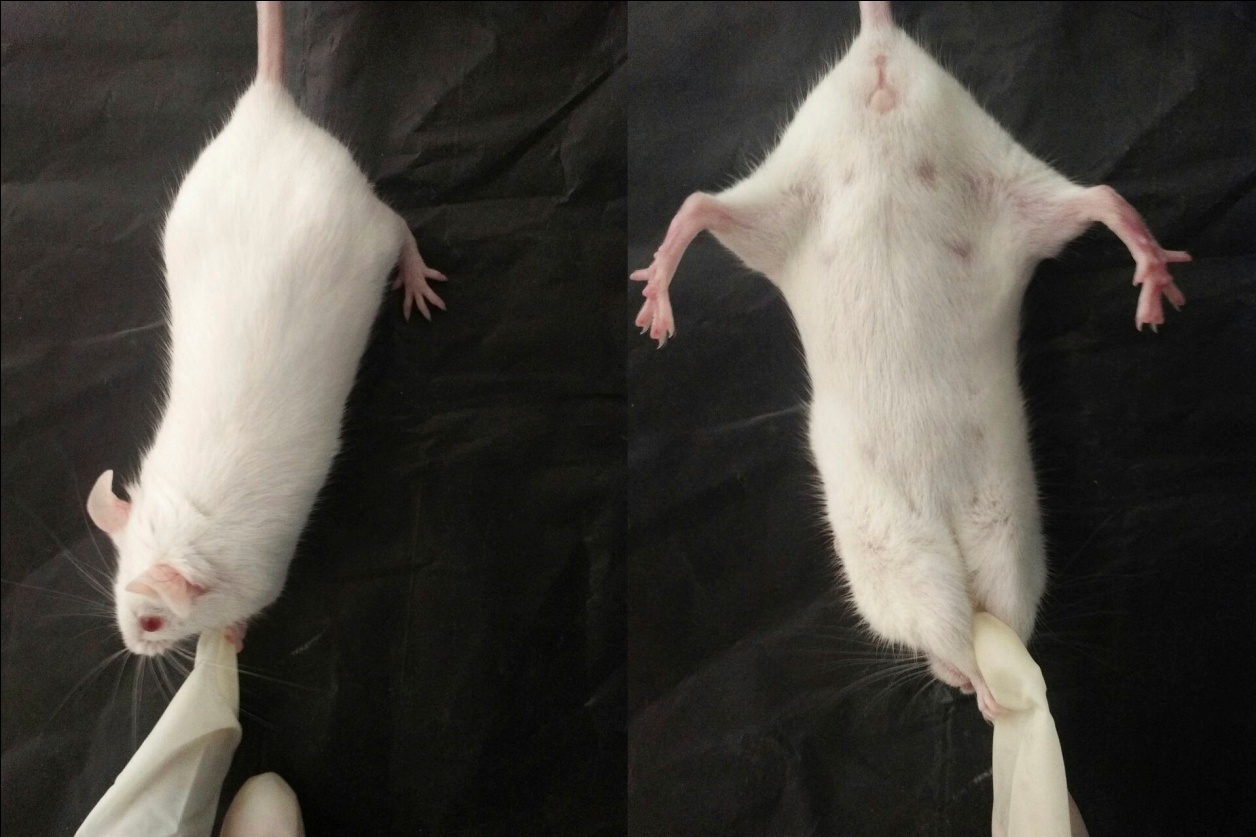


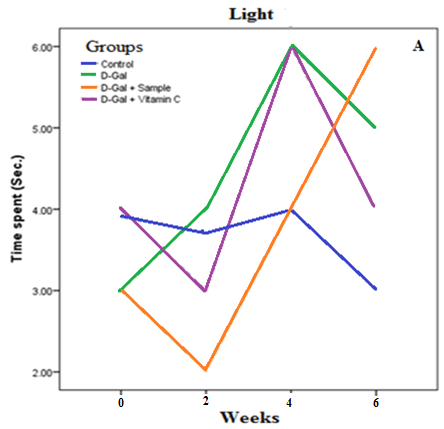

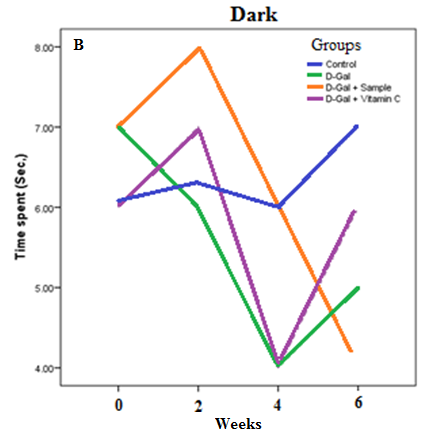


**Fig. S1 (A&B). Light & dark activities of experimental mice**

**Fig. S2. Control Mice (Group I)**


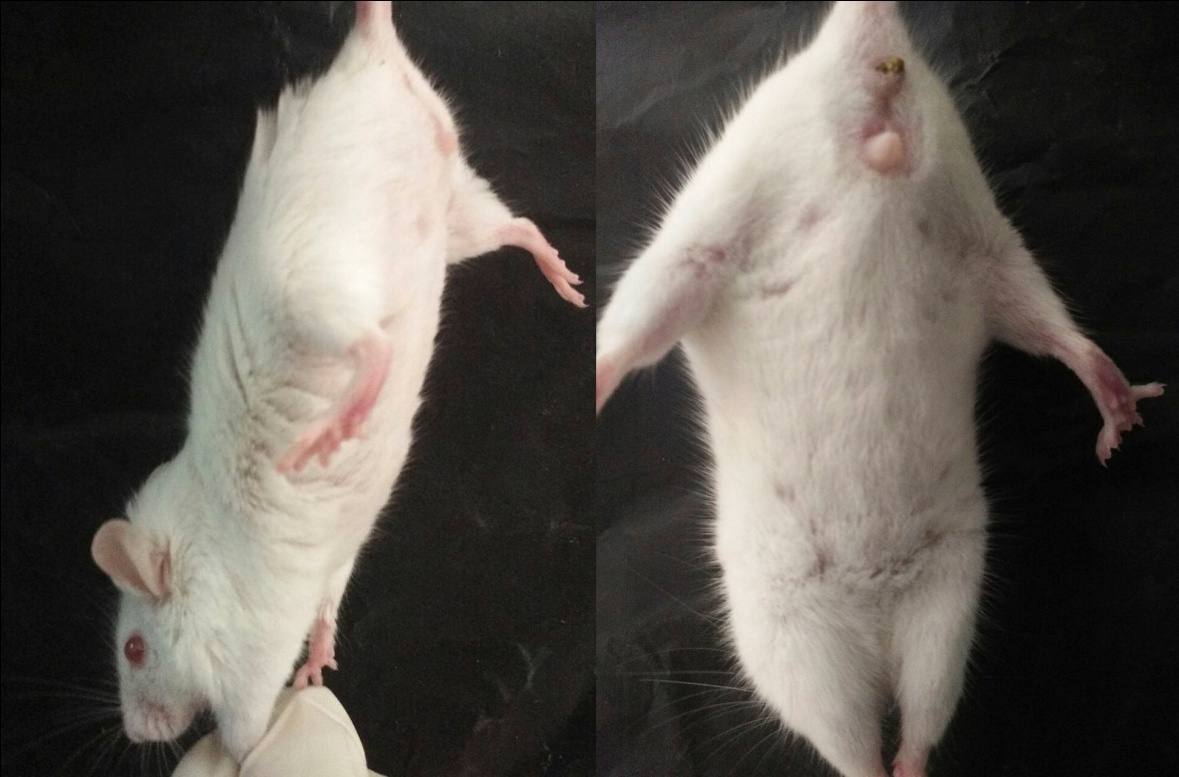


**Fig. S3. Agar treated (Group III) mice**


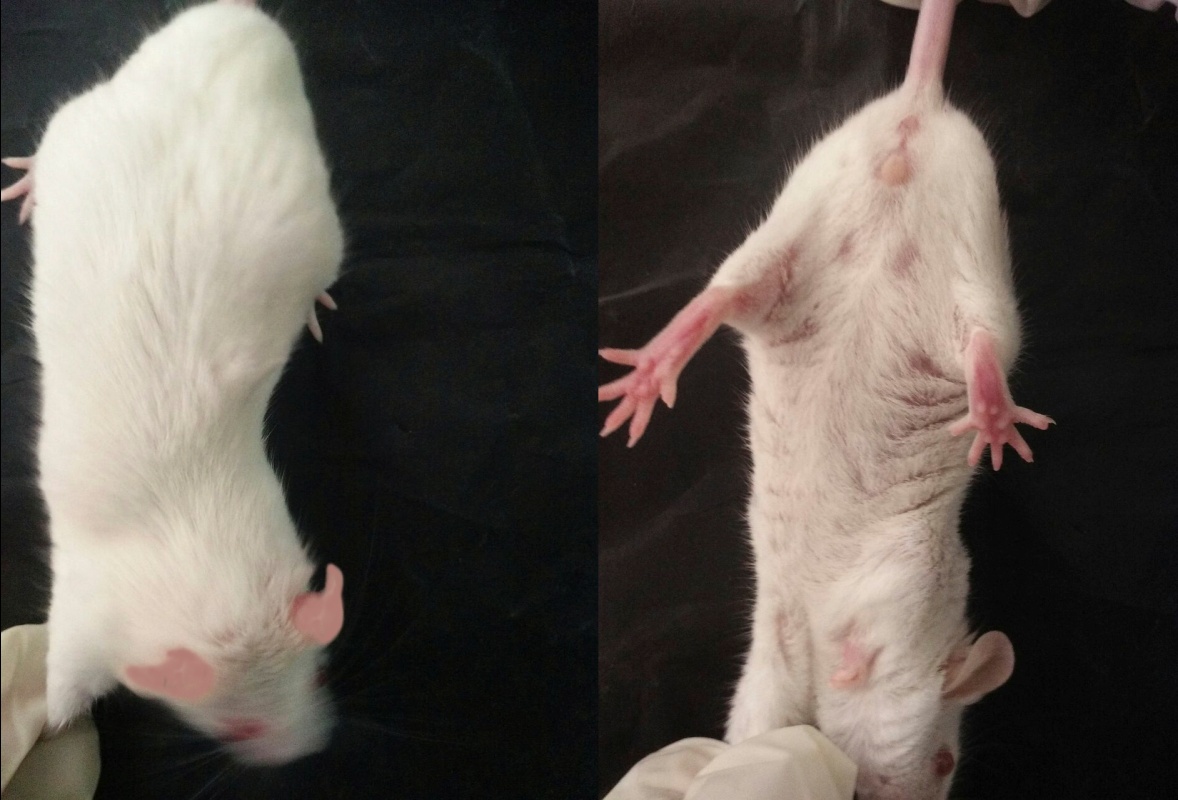


**Fig. S4. D-Gal treated (Group II) Mice (2^nd^ weeks)**
